# Parental Factors Related to Physical Activity among Adolescent Men Living in Built and Natural Environment: A Population-Based MOPO Study

**DOI:** 10.1155/2021/3234083

**Published:** 2021-05-24

**Authors:** Riitta Pyky, Soile Puhakka, Tiina M. Ikäheimo, Tiina Lankila, Maarit Kangas, Matti Mäntysaari, Timo Jämsä, Raija Korpelainen

**Affiliations:** ^1^Department of Sports and Exercise Medicine, Oulu Deaconess Institute Foundation sr., Albertinkatu 18A, Oulu 90100, Finland; ^2^Center for Life Course Health Research, Faculty of Medicine, University of Oulu, P.O. Box 8000, Oulu FI-90014, Finland; ^3^Research Unit of Medical Imaging, Physics and Technology, Faculty of Medicine, University of Oulu, P.O. Box 8000, Oulu FI-90014, Finland; ^4^Geography Research Unit, P.O. BOX 8000, University of Oulu, Oulu FI-90014, Finland; ^5^Medical Research Center Oulu, Oulu University Hospital and University of Oulu, P.O. Box 8000, Oulu FI-90014, Finland; ^6^Center for Environmental and Respiratory Health Research, University of Oulu, P.O. Box 5000, Oulu FI-90014, Finland; ^7^Centre for Military Medicine, The Finnish Defense Forces, P.O. Box 50, Tukholmankatu 8 A, Helsinki FI-00301, Finland; ^8^Department of Diagnostic Radiology, Oulu University Hospital, P.O. Box 50, Oulu 90029, Finland

## Abstract

**Introduction:**

Physical inactivity is a global concern, especially among adolescent men. Little research has been done on the association between parental factors and young adults' physical activity in the context of residential environment. We aimed to reveal what parental factors are associated with physical activity among adolescent men living in built and natural environments.

**Methods:**

A population-based sample of 1,904 men (mean age = 17.9, SD = 0.7 years) completed a questionnaire regarding physical activity, parental factors, and lifestyle in Northern Finland in 2012 and 2013. Geographical information system methods and dominant land-use type were used to define the residential environment in a 1-kilometer radius buffer zone surrounding each participant's home address. If the residential area included more artificial surfaces, it was defined as a built environment, and areas including more nature were defined as natural environments.

**Results:**

According to multivariable analyses, a mother's physical activity (OR = 1.9; 95% CI: 1.3–2.8) was positively associated with the physical activity of adolescent men living in built environments, and the father's physical activity was positively associated with the physical activity of adolescent men living in natural environments (2.8; 1.7–4.8). Self-rated health (built 5.9 [4.0–8.7]; natural 5.2 [3.0–9.0]) was positively associated with physical activity level. Those with symptoms of depression were more likely to be physically inactive (built 0.5 [0.3–0.8]; natural 0.3 [0.1–0.6]). Adolescent men were equally physically active regardless of the living environment.

**Conclusions:**

The level of physical activity of parents, self-rated health, and depressive symptoms should be considered when designing physical activity promotions for adolescent men according to their residential environments.

## 1. Introduction

Physical activity (PA) promotes both physical and mental health by, for example, reducing coronary heart disease and depression [1]. However, only 20% of adolescents worldwide and in Finland are physically active enough to achieve the health benefits of PA [2, 3]. Physical activity is also of high importance in decreasing the health risks induced by sedentary behavior [4]. Adolescents spend approximately six to eight hours daily on sedentary behaviors [5, 6], but Finnish young men may sit for more than 10 hours daily [7]. Although boys meet the PA recommendation more often than girls in childhood, the decline in PA has been rapid in both genders, both globally [8] and in Finland [9]. The decline may be steeper among boys than girls, despite boys' higher activity in childhood [10].

Healthy young men may engage in risky behaviors such as physical inactivity and unhealthy diets, placing themselves at risk for impaired health in the future [11]. Physical inactivity and other unhealthy behaviors seem to accumulate among Finnish young men [12–15]. Boys and young men have been reported to often be more overweight compared to Finnish girls [16] and to spend more time on sedentary behaviors than their coevals globally [5–7].

The determinants of PA among young people are not fully known. Environmental factors, such as the amount of green space and the density of pedestrian route networks, were recently suggested to be positively associated with PA [17]. In a recent review on diet and PA in rural versus urban children and adolescents, the majority of studies assessing PA reported either no differences or findings that indicated rural youth were more physically active [18]. However, the results were inconsistent, and there were strong methodological disparities between the studies. The authors also pointed out that a factor that may have played a role could be team sport participation because, in rural locations where there might be only 10 to 20 students in a particular grade, a majority of students would be required for a sports team to have enough players, and many youths participate in every sport offered. Among children, significant interactions between home and parental factors and PA according to urban/rural locations have been suggested [19].

In addition, some evidence suggests that parental factors, such as supporting and participating in adolescents' physical activities, are of high importance [20, 21]. Earlier studies suggested that parents' PA and adolescents' perceptions of their parents' PA may not be related to PA in youths [21], but a systematic association between parents' PA and the PA of their offspring was found from childhood to young adulthood in a Finnish prospective and population-based study [22]. In addition to support and role modeling, parents can organize and transport children to sport activities, as well as encourage and follow their child's sport activities and coparticipate PA with their child [23]. Fathers may play a more important role in their children's leisure-time activities in Finnish rural areas [24]. Moreover, several mental health problems, for example, depression, may have an effect on the PA of adolescents. However, males who exercise more perceive themselves as healthier and report fewer depressive symptoms [25, 26]. Physical activity level and willingness to be physically active have also been closely linked to self-rated health [25, 27].

Features of natural and built environments may be associated with PA [17]. Built environments include structures and spaces constructed and modified by humans, such as streets, sidewalks, bicycle lanes, transportation systems, and buildings. The natural environment is composed of ecosystem resources, such as climate, vegetation, forests, fields, and so forth [28]. Environmental determinants of PA vary depending on the type of PA studied. Previous studies on the association between residential environments and PA have mainly focused on built and urban environments, although green areas have been shown to promote PA, especially moderate to vigorous PA, and self-rated health [29–31]. Key features of built environments in promoting PA include a dense population, high intersection and public transport density, and a high number of parks [17]. Moreover, the type of PA may vary according to the type of green space: greenness in a built environment encourages more walking, while agricultural environments foster gardening and other habitual chores [32]. Long distances to sport facilities, lack of services, and poor transportation can be barriers to PA among youth in rural environments, but natural amenities are seen as resources for PA [33]. Little is known about the type of, motives for, and amount of youths' PA in natural environments.

The majority of previous studies on the relationship between the environment and PA have been conducted on adults, whereas only a few studies involving young people exist, and the findings are contradictory [34–36]. Adolescents spend little time in green spaces, with boys spending a little more time than girls [31]. To urban adolescents, streets and school grounds are more important PA facilities than city parks [31, 32, 37], but findings [38, 39] on the use of city green spaces are also inconsistent. Rural young people are more likely to use farmland and grassland than urban youth. Domestic gardens seem to be potential places for both light and vigorous activity. However, previous studies have mainly been based on the perceived environment. [21, 31].

In summary, the influence of parental behavior on youths' PA is heterogeneous, and information on the role of environmental features in young people's PA is scanty or focused mainly on built environments and urban areas. Some previous studies have examined the relationship between parental PA and adolescents' PA [21, 35, 36, 40], but only a few of these considered the residential environment as a modifying factor.

Because the factors underlying youths' PA are diverse and they might be environmentally and culturally dissenting, the aim of this study was to explore PA among youth and the parental correlates of PA taking into account self-rated health and depressive symptoms among adolescent men living in either built or natural environments. Our hypothesis was that adolescent men living in built environments are physically more active than those living in natural environments. We also expected that the parental correlates of PA are different in natural versus built environments.

## 2. Materials and Methods

This cross-sectional study is part of a population-based study (MOPO) that aims to promote PA and to prevent social marginalization among young, conscription-aged men [41]. Military service or civic duty is compulsory for all male citizens of Finland, and the Finnish Defense Forces organizes conscription every year for males who turn 18 in that year. The entire age cohort attends the conscription except those whose health or psychological capacities do not allow independent living. Thus, conscription provides a large, population-based representative sample of adolescent men.

All men who attended the conscription for military service in the Oulu area of Northern Finland in September 2012 and September 2013 (*N* = 2,547) were asked to complete a questionnaire inquiring about PA, health, and lifestyle. A total of 1,904 (74.8%) men (mean age 17.9, SD = 0.7 years) completed the questionnaire. Their height and weight were measured.

### 2.1. Questionnaire

Participants reported their highest educational level, from basic education only to vocational upper secondary school, general upper secondary school, or higher education.

Leisure-time PA (LTPA) was assessed with the frequently used question “How much do you exercise or strain yourself physically in your leisure time?” [42]. Response options were (1) I read, watch TV, and do light housework; (2) I walk, cycle, or otherwise exercise for at least four hours per week (excluding travel to work or school); (3) I exercise to maintain my physical condition by, for example, running, jogging, cross-country skiing, doing gymnastics, swimming, playing ball games, or doing heavy gardening for at least two hours weekly; and (4) I take part in competitive sports or other heavy exercise several times a week. The first response option was categorized as low LTPA. Categories 2 to 4 were pooled together and renamed high LTPA. The dichotomous variable (1 vs. 2–4) was used as a dependent variable in analyses.

Barriers to PA were studied on a 2-point scale: “Is tiredness due to work or school/lack of time/lack of appropriate exercise group/laziness/poor transportation restricting your physical activity?” (yes/no). Participants also evaluated whether enhancing health or physical fitness or enjoying the feelings of euphoria gained via exercise motivated them to exercise (yes/no) [43]. The participants' PA types were inquired with an open question and categorized by the researcher as ball games, ice hockey, gym, running/jogging/walking/cycling, martial arts, and other types of exercise (e. g., downhill skiing, roller skating, snowboarding, athletics, bowling, and extreme sports).

The participants rated their health as good, reasonably good, average, reasonably poor, or poor. Depressive symptoms were evaluated with Raitasalo's modification of the Beck Depression Inventory (BDI), which has been shown to have high validity compared to the unmodified BDI [44]. The respondents were asked whether they currently smoked. A question also asked about the frequency of binge drinking at least six servings of alcohol at a time (none, less than once a month, once a month, once a week, two or three times a week, or daily or almost daily).

The participants reported their parents' occupations with an open-ended question. Occupations were categorized according to Classification 89 of Occupations 2010 by Statistics Finland (2013). The categories were further divided into six subcategories as follows: (1) managers and entrepreneurs, (2) professionals, (3) associate professionals, (4) service and sales workers, (5) manual laborers, and (6) others (unemployed, pensioner, student, or unknown). Categories 1 to 3 and 4 to 6 were grouped together and used as dichotomous variables in the analyses.

The participants evaluated their parents' PA with the following question: “How regularly is your mother/father physically active?” The answer options were (1) daily, (2) several times a week, (3) a few times a month, (4) hardly ever, and (5) I do not know. Options 1 and 2 were classified as parent(s) being physically active. In addition, participants reported whether they had exercised with their parent(s) while they were primary school-aged (7–12 years old) and junior high school-aged (13–15 years old).

### 2.2. Residential Environment

Geographical information system (GIS) methods were used to define the dominant land-use type of each participant's residential environment. ArcMap 10.2 software (Environmental Systems Research Institute [ESRI], 2014, ArcGIS Desktop 10.2, Redlands, California, USA) and the Tabulate Overlapping Polygons tool were used to create a circular 1-kilometer buffer zone around each participant's home address (coordinate point, EUREFIN). Land use could fall into five categories, namely, (1) artificial surfaces, (2) agricultural areas, (3) forests and seminatural areas, (4) wetlands, and (5) water bodies, and was calculated within the buffer zone with square meter accuracy. The category that covered the largest share of the buffer zone determined the land-use type of the participant's residential environment. Category 1 represents built residential environments, and categories 2, 3, 4, and 5 represent natural residential environments. Natural environments include both rural areas and areas dominated by nature. The land use is based on CORINE Land Cover data (one grid 20 m × 20 m), which is spatial information on land cover obtained from satellite data.

### 2.3. Statistical Analysis

The statistical significance of the differences between the adolescent men living in built and natural environments was analyzed using the chi-square test for the categorical variables and the independent samples *t*-test for the continuous variables.

For the sample, we evaluated that the proportion of inactive participants is 20% in natural environments and 15% in built environments. With a study power of 80% and significance level of 5%, the calculated sample size was 906 per group. To evaluate the factors associated with self-rated PA separately for participants living in natural versus built environments, we conducted multivariable binary logistic regression analyses with the enter method. Variables associated with PA in univariate analyses (separate for the two environments, including self-rated health and depressive symptoms) and without multicollinearity were entered into the logistic regression analysis. The level of significance was set at *p* < 0.07 to avoid excluding any variables potentially associated with reported PA. Nonsignificant variables were removed from the model one by one. The results are presented as odds ratios (ORs) with 95% confidence intervals (CIs). Statistical significance was set at *p* < 0.05. The data were analyzed with PASW Statistics software (IBM Corp. Released 2013. IBM SPSS Statistics for Windows, Version 22.0. Armonk, NY: IBM Corp.).

## 3. Results

The characteristics of the study participants are presented in Table 1. Altogether 1,144 (62%) adolescent men lived in built environments and 714 in natural environments. Eighty percent of the built environment participants and 87% of the natural environment participants were currently living with their parent(s). Currently living with parents was significantly correlated with exercising with parents. Therefore, this latter variable was chosen for the analyses. The participants living in natural environments had attended vocational upper secondary school more often than those living in built environments (*p*=0.003 for the difference between the groups). Built environment residents used alcohol more frequently (*p*=0.045). There was a statistically significant (*p*=0.029), but practically nonmeaningful, 1.2-month age difference between the participants living in natural and built environments. There were no statistical differences in self-rated PA, parental PA, or occupation according to the residential environment of the participants.

Motives for PA did not differ between the participants living in built or natural environments. Health promotion (built 82.5% and natural 83.6% reporting this motive), enjoying the feelings of euphoria gained via exercise (both groups 75.5%), and fitness enhancement (built 75.1%; natural 75.3%) were the most common motives for exercise in both environment types. In addition, participants in both groups often reported tiredness (built 49.1%; natural 51.5%), laziness (both groups 42.4%), or lack of time (built 33.4%; natural 33.9%) as restricting their PA. Poor transportation (*p*=0.002) and lack of appropriate exercise group (*p*=0.04) restricted residents of natural environments more than those residing in built environments. The most popular types of exercise were ball games (built 22.7%; natural 24.0%); going to the gym (built 26.4%; natural 21.9%); and walking, jogging, or cycling (built 18.7%; natural 17.5%).

The factors associated with self-rated LTPA in the residential environment group in the univariate analysis are presented in Table 2. Education level, weight, mother's occupation, and exercising with parents at primary school age were associated with PA among residents of built but not natural environments. However, father's occupation was associated with PA in participants living in natural but not built environments.

According to the binary logistic regression (Table 3) by residential environment, self-perceived health (OR = 5.9; 95% CI: 4.0–8.7) and mother's PA (OR = 1.9; 95% CI: 1.3–2.8) were significantly and positively associated with PA in participants living in built environments. In contrast, self-rated health (OR = 5.2; 95% CI: 3.0–9.0) and father's PA (OR = 2.8; 95% CI: 1.7–4.8) were significantly associated with PA in participants living in natural environments. Those with symptoms of depression were more likely to be physically inactive (built: OR = 0.5; 95% CI: 0.3–0.8 and natural: OR = 0.3; 95% CI: 0.1–0.6).

## 4. Discussion

In this population-based study, we compared the correlates of leisure-time PA, with special emphasis on parental factors, among adolescent men living in built and natural environments in Finland. Mothers' PA was positively associated with the PA of participants living in built environments, whereas fathers' PA was emphasized among natural environment residents. Self-rated health and depressive symptoms were significantly associated with PA in both environments.

This is the first large-scale study to reveal differences in associations between parents' and their children's PA according to the residential environment. In our study, the parental roles related to PA differed according to residential area. Although it has been suggested that the support of parents is of great importance to the PA of adolescents [20], previous studies have revealed a heterogeneous or even lacking association between the PA of parents and their children [21]. In two comprehensive studies involving adolescents from Brazil and urban youths from Spain, fathers' PA was associated with boys' self-rated PA. [35, 36] However, rural and urban residents were not compared as in our study. Mothers' PA has also been found to be associated with the PA of boys in earlier studies [45], although it has more commonly been associated with the PA of girls [46]. In a South Indian study, in contrast to our study, no difference according to the type of residential environment was detected regarding the association of PA of parents and adolescents. This may partly be due to different definitions of the participants' residential area. In this study, we used dominant land-use type, whereas population density, distance to city centers, and percentage of the population employed in agricultural work were used in the Indian study [34]. Other Finnish studies have also detected that fathers play a central role in leisure-time activities among rural adolescents [24]. A recent Finnish study showed that if mothers and fathers are highly physically active, this may increase their children's PA up to young adulthood, despite the residential environment or parents' socioeconomic status. Higher physical activity of fathers was associated with their sons' PA, while physically active mothers may increase their daughters' PA. After adjusting for covariates, mothers' PA was especially associated with their sons' PA. [22] Salmon et al. did not find differences according to residential environment as we did in our study. An Australian study reported that mothers in rural areas have more physical activity equipment at home and their social network and neighborhood support children's physical activity [19]. Fathers were not included in the Australian study. In Finland, parents may have different roles in the family according to the residential environment. In rural and more natural areas, fathers may spend more time with their sons. In built and urban areas, mothers may be responsible for taking their children to hobbies, and mothers who are physically active themselves presumably support their children more in being physical active. According to the Statistics of Finland, the amount of single fathers of all families was higher in rural compared to urban areas, which may have affected on our results [47]. Ultimately, the varying parental roles and, for example, differences across countries may be due to complex cultural factors, such as gender roles, environmental factors, and youth culture, making it difficult to distinguish the underlying reasons or to make comparisons with other studies.

According to our findings and consistent with previous studies, self-rated health is associated with the PA of adolescent men irrespective of the residential area. The presence of and access to green spaces in the suburbs have previously been found to be related to LTPA and self-rated health in a random sample of 8,000 Finns [48]. Mental health and PA are strongly related among adolescents [26], which agrees with the findings in this study, with negative associations between depressive symptoms and PA in both built and natural residential environments.

Motives for and restrictions on PA were otherwise similar in different residential environments, except that poor transportation and a lack of appropriate exercise groups restricted PA more among residents in natural environments. Because the natural residential environment category included participants living in rural areas, this finding is not surprising. Public transportation levels and guided exercise possibilities are usually limited in rural, and even suburban, environments compared to urban areas.

The participants were equally physically active regardless of the residential environment. Objectively measured moderate to vigorous PA has previously been shown to be lower in rural youth compared to urban youth [49]. However, differences in PA according to the residential environment have not always been detected among boys [50], which is consistent with this study. Higher moderate to vigorous PA and less sedentary time have been found in less densely populated urban areas when compared to more densely populated urban areas among adolescents in the United States [51]. Differences between the results may be due to the methods used to define behavior or the residential environments or differences in the surrounding green spaces.

Previous studies on young people have defined the residential environment as rural, suburban, or urban, based mainly on adjacency to centers or number of inhabitants [35, 49]. To our knowledge, we are the first to use dominant land-use type to categorize the residential environment. Natural residential environments include rural environments, but also reflect the number of natural elements and the quality of the participant's residential area. From a resident's perspective, a relevant issue for PA is not only the adjacent city centers or density of population, but also the environmental quality and the features offered by the everyday residential environments [52, 53]. Employing the land-use type for categorizing the residential environment provides more information regarding the quality and features of the environment. Instead of mere population density, these features provide additional insight when studying how environments affect PA. Dominant land-use type is one measure of environmental quality, but a direct comparison between our study and the previous studies is not possible due to the different methods of characterizing these environments.

The strength of this study is in its population-based design and objectively measured built and natural residential environments. One limitation is that we did not know how long the participants had been living in their current residential areas. There might be a recall bias concerning the measure of exercising with parents in childhood. As parental PA was evaluated by the participants, not by the parents themselves, this may bias our results. The absence of female adolescents in the sample may also be considered a limitation, and the results are not applicable to female populations. However, the annual conscription is only organized for men, and it was not possible to recruit a similar population-based sample of female adolescents. A methodological limitation is the use of the PA questionnaire. There are common well-known errors (recall and social desirability bias) inherent in self-reporting of PA [54]. Physical activity questionnaires tend to overestimate the true PA levels but can provide essential information regarding the type and context of PA [55]. Because of the cross-sectional study design, conclusions about causality of the results cannot be drawn.

The results of the present study can be utilized in targeting and tailoring preventive actions for young adults in Western countries and especially in Northern Europe. Our study adds knowledge on the parental and environmental factors associated with the PA of young men and possible residential differences in PA among youth. Finally, the PA patterns of the parents and other characteristics related to the residential environment should be considered when examining the PA of young men.

## 5. Conclusions

In this population-based study of adolescent men, mothers' PA was associated with the PA of adolescent men living in built environments, while fathers' PA was associated with the PA of those living in natural environments. Self-rated health and depressive symptoms were associated with PA in both environments. This study adds to the literature on individual, environmental, and parental factors underlying PA in young men. These findings highlight the importance of individually designed health promotion for young men.

## Figures and Tables

**Table 1:** Characteristics of the participants (*n* = 1,904) according to their residential environment.

Variable	All (*n* = 1,904)	Men living in built environment (*n* = 1,144)	Men living in natural environment (*n* = 714)	*p* ^a^
Age, years, mean (*SD*)	17.9 (0.7)	17.9 (0.7)	17.8 (0.7)^*∗∗*^	**0.029**
Education, *N* (%)			^*∗*^	**0.003**
Primary school	112 (6.0)	69 (6.2)	33 (4.7)	
Vocational school	823 (44.2)	462 (41.2)	343 (49.1)	
Upper secondary school or higher	927 (49.8)	590 (52.6)	322 (46.1)	

Living currently at home, *N* (%)	1,556 (81.9)	908 (79.5)	623 (87.3)	**< 0.001**
Weight, kg, mean (*SD*)	73.0 (14.7)	73.5 (15.2)	72.4 (13.8)	0.140
Height, cm, mean (*SD*)	177.7 (6.4)	177.7 (6.5)	177.9 (6.3)	0.733
BMI, kg/m^2^, mean (*SD*)	23.1 (4.4)	23.2 (4.5)	22.9 (4.1)	0.087
Leisure-time physical activity, *N* (%)				0.111
I read, watch TV, and do light housework.	341 (18.4)	215 (19.2)	115 (16.6)	
I walk, cycle, or otherwise exercise at least four hours per week (excluding travel to work or school).	564 (30.4)	325 (29.1)	227 (32.7)	
I exercise to maintain my physical condition at least two hours weekly.	682 (36.7)	402 (36.0)	262 (37.8)	
I take part in competitive sports or other heavy sports several times a week.	269 (14.5)	175 (15.7)	90 (13.0)	

RBDI score^b^, mean (SD)	1.6 (3.2)	1.6 (3.1)	1.5 (3.2)	0.802
Self-rated health; good or reasonably good, *N* (%)	1,329 (73.8)	791 (72.8)	507 (75.3)	0.265
Physically active mother, *N* (%)	1,103 (69.3)	654 (68.9)	424 (69.6)	0.779
Physically active father, *N* (%)	995 (65.8)	575 (64.6)	397 (67.5)	0.263
Current smoker, *N* (%)	505 (26.9)	302 (26.8)	188 (26.7)	0.989
Alcohol binge drinking ≥ once a week, *N* (%)	315 (17.7)	205 (19.2)	102 (15.4)	**0.045**
Father's occupation: manager, entrepreneur, professional, associate professional, *N* (%)	747 (49.3)	444 (50.0)	287 (48.9)	0.710
Mother's occupation: manager, entrepreneur, professional, associate professional, *N* (%)	798 (50.8)	467 (49.7)	314 (52.5)	0.296
Exercised with parents while elementary school-aged *N* (%)	1,238 (71.5)	732 (70.0)	477 (74.0)	0.086
Exercised with parents while secondary school-aged *N* (%)	464 (27.4)	282 (27.6)	178 (28.1)	0.866

Numbers do not match due to missing values. *p*^a^ = chi-square test or independent samples *t*-test for the difference between the participants living in built or natural environments. ^b^Finnish modification of the short form of the Beck Depression Inventory; the lower the value, the fewer the depression symptoms, scale 0–39.  ^*∗*^*p* < 0.05;  ^*∗∗*^*p* < 0.01;  ^*∗∗∗*^*p* < 0.001, reference group: all. BMI: body mass index; RBDI: Raitasalo's Beck Depression Index.

**Table 2:** Univariate associations between the explanatory variables and leisure-time physical activity (LTPA) among the participants living in built and natural environments.

Variable	Built environment (*n* = 1,144)		*p* ^a^	Natural environment (*n* = 694)		*p* ^a^
	Low LTPA (*n* = 215)	High LTPA (*n* = 902)		Low LTPA (*n* = 115)	High LTPA (*n* = 579)	
	*n* (%)	*n* (%)		*n* (%)	*n* (%)	
Education: basic education or vocational upper secondary school	117 (56.0)	399 (45.1)	**0.005**	70 (61.4)	293 (52.1)	0.080
Weight, kg, mean (SD)	76.1 (21.5)	73.2 (13.9)	**0.047**	73.5 (17.5)	72.0 (12.3)	0.366
At least mild depression symptoms	47 (22.7)	61 (7.1)	**<0.001**	34 (30.9)	36 (6.7)	**<0.001**
Self-rated health: good or reasonably good	82 (39.6)	698 (81.1)	**<0.001**	45 (41.3)	453 (82.1)	**<0.001**
Father's occupation: associate professional or higher	68 (44.2)	367 (51.2)	0.131	34 (36.6)	250 (51.8)	**0.009**
Mother's occupation: associate professional or higher	64 (38.8)	394 (52.3)	**0.002**	46 (46.9)	262 (53.8)	0.224
Exercised with parents in primary school	121 (62.4)	603 (72.1)	**0.009**	74 (67.3)	397 (75.6)	0.073
Exercised with parents in junior high school	36 (19.0)	243 (29.8)	**0.003**	17 (15.7)	159 (30.8)	**0.002**
Physically active father	74 (50.3)	495 (67.7)	**<0.001**	42 (49.4)	350 (71.0)	**<0.001**
Physically active mother	90 (55.6)	555 (71.6)	**<0.001**	54 (57.4)	362 (72.0)	**0.005**

Values are *N* (%) unless otherwise stated. *p*^a^ = independent samples *t*-test for continuous variables and Pearson chi-square test for categorical variables for the difference between the low and high LTPA groups in the residential environments. LTPA: self-rated leisure-time physical activity; low LTPA: light housework but no other LTPA; high LTPA: physical activity or exercising several times weekly.

**Table 3:** Factors associated with high self-rated leisure-time physical activity (PA) (OR, 95% CI) among built and natural environment residents according to the multivariable binary logistic regression analysis.

Variable	Adjusted OR (95% CI)	*p*
Natural environment, *n* = 553, model *R*^2^ = 0.249		
Quite good or better self-rated health (vs. moderate, poor, or extremely poor)	5.2 (3.0–9.0)	<0.001
Mild depression symptoms or higher (vs. none or only extremely mild symptoms)	0.3 (0.1–0.6)	<0.001
Father exercises at least several times a week (vs. few times a month or almost never)	2.8 (1.7–4.8)	<0.001

Built environment, *n* = 903, model *R*^2^ = 0.207		
Quite good or better self-rated health (vs. moderate, poor, or extremely poor)	5.9 (4.0–8.7)	<0.001
Mild depression symptoms or higher (vs. none or only extremely mild symptoms)	0.5 (0.3–0.8)	0.010
Mother exercises at least several times a week (vs. few times a month or almost never)	1.9 (1.3–2.8)	0.001

OR: odds ratio; CI: confidence interval; according to multivariable logistic regression analyses. Model *R*^2^: Nagelkerke regression coefficient; PA: physical activity.

## Data Availability

Data are available from the University of Oulu to research collaborators who meet the criteria for accessing confidential data. Contact the project center for further information: projectcenter@oulu.fi.

## Abbreviations

BDI: Beck's Depression Inventory
CI: Confidence interval
GIS: Geographical information system
LTPA: Leisure-time physical activity
MET: Metabolic equivalent
MVPA: Moderate to vigorous physical activity
OR: Odds ratio
PA: Physical activity.

## References

[1] Lee I.-M., Shiroma E. J., Lobelo F. (2012). Effect of physical inactivity on major non-communicable diseases worldwide: an analysis of burden of disease and life expectancy. *The Lancet*.

[2] Hallal P. C., Andersen L. B., Bull F. C. (2012). Global physical activity levels: surveillance progress, pitfalls, and prospects. *The Lancet*.

[3] Kämppi K., Aira A., Halme N. (2018). Results from Finland’s 2018 report card on physical activity for children and youth. *Journal of Physical Activity and Health*.

[4] Ekelund U., Steene-Johannessen J., Brown W. J. (2016). Does physical activity attenuate, or even eliminate, the detrimental association of sitting time with mortality? a harmonised meta-analysis of data from more than 1 million men and women. *The Lancet*.

[5] Fröberg A., Raustorp A. (2014). Objectively measured sedentary behaviour and cardio-metabolic risk in youth: a review of evidence. *European Journal of Pediatrics*.

[6] Pate R. R., Mitchell J. A., Byun W., Dowda M. (2011). Sedentary behaviour in youth. *British Journal of Sports Medicine*.

[7] Niemelä M., Ahola R., Pyky R. (2016). Self-reported versus measured physical activity and sedentary behavior in young men. *Liikunta & Tiede*.

[8] Cooper A. R., Goodman A., Page A. S. (2015). Objectively measured physical activity and sedentary time in youth: the International children’s accelerometry database (ICAD). *International Journal of Behavioral Nutrition and Physical Activity*.

[9] Liukkonen J., Jaakkola T., Kokko S. (2014). Results from Finland’s 2014 report card on physical activity for children and youth. *Journal of Physical Activity and Health*.

[10] Corder K., Sharp S. J., Atkin A. J. (2015). Change in objectively measured physical activity during the transition to adolescence. *British Journal of Sports Medicine*.

[11] Ashton L. M., Hutchesson M. J., Rollo M. E., Morgan P. J., Collins C. E. (2014). A scoping review of risk behaviour interventions in young men. *BMC Public Health*.

[12] Santtila M., Pihlainen K., Koski H., Vasankari T., Kyröläinen H. (2018). Physical fitness in young men between 1975 and 2015 with a focus on the years 2005–2015. *Medicine & Science in Sports & Exercise*.

[13] Kantomaa M. T., Tammelin T. H., Ebeling H. E., Taanila A. M. (2008). Emotional and behavioral problems in relation to physical activity in youth. *Medicine & Science in Sports & Exercise*.

[14] Pohjola V., Nurkkala M., Virtanen J. I. (2021). Psychological distress, oral health behaviour and related factors among adolescents: finnish school health promotion study. *BMC Oral Health*.

[15] Bingham C. M., Jallinoja P., Lahti-Koski M. (2010). Quality of diet and food choices of Finnish young men: a sociodemographic and health behaviour approach. *Public Health Nutrition*.

[16] Jääskeläinen S., Mäki P., Mölläri K., Mäntymaa P. (2020). *Overweight and Obesity in Children and Adolescents 2019: Every Fourth Boy and Almost Every Fifth Girl was Overweight or Obese*.

[17] Sallis J. F., Cerin E., Conway T. L. (2016). Physical activity in relation to urban environments in 14 cities worldwide: a cross-sectional study. *The Lancet*.

[18] McCormack L. A., Meendering J. (2016). Diet and physical activity in rural vs urban children and adolescents in the United States: a narrative review. *Journal of the Academy of Nutrition and Dietetics*.

[19] Salmon J., Veitch J., Abbott G. (2013). Are associations between the perceived home and neighbourhood environment and children’s physical activity and sedentary behaviour moderated by urban/rural location?. *Health & Place*.

[20] Sleddens E. F. C., Kremers S. P. J., Hughes S. O. (2012). Physical activity parenting: a systematic review of questionnaires and their associations with child activity levels. *Obesity Reviews*.

[21] Bauman A. E., Reis R. S., Sallis J. F. (2012). Correlates of physical activity: why are some people physically active and others not?. *The Lancet*.

[22] Kaseva K., Hintsa T., Lipsanen J. (2017). Parental physical activity associates with offspring’s physical activity until middle age: a 30-year study. *Journal of Physical Activity and Health*.

[23] Hutchens A., Lee R. E. (2018). Parenting practices and children’s physical activity: an integrative review. *The Journal of School Nursing*.

[24] Tuuva-Hongisto S., Pöysä V., Armila P. (2016). *Young People in Remote Villages - Forgotten Residents?*.

[25] Herman K. M., Hopman W. M., Sabiston C. M. (2015). Physical activity, screen time and self-rated health and mental health in Canadian adolescents. *Preventive Medicine*.

[26] Biddle S. J. H., Asare M. (2011). Physical activity and mental health in children and adolescents: a review of reviews. *British Journal of Sports Medicine*.

[27] Kantomaa M. T., Tammelin T., Ebeling H., Stamatakis E., Taanila A. (2015). High levels of physical activity and cardiorespiratory fitness are associated with good self-rated health in adolescents. *Journal of Physical Activity and Health*.

[28] Borchardt J. L., Paulitsch R. G., Dumith S. C. (2019). The influence of built, natural and social environment on physical activity among adults and elderly in southern Brazil: a population-based study. *International Journal of Public*.

[29] James P., Banay R. F., Hart J. E., Laden F. (2015). A review of the health benefits of greenness. *Current Epidemiology Reports*.

[30] Dadvand P., Bartoll X., Basagaña X. (2016). Green spaces and general health: roles of mental health status, social support, and physical activity. *Environment International*.

[31] McCrorie P. R., Fenton C., Ellaway A. (2014). Combining GPS, GIS, and accelerometry to explore the physical activity and environment relationship in children and young people - a review. *International Journal of Behavioral Nutrition and Physical Activity*.

[32] Picavet H. S. J., Milder I., Kruize H., de Vries S., Hermans T., Wendel-Vos W. (2016). Greener living environment healthier people?. *Preventive Medicine*.

[33] Edwards M. B., Theriault D. S., Shores K. A., Melton K. M. (2014). Promoting youth physical activity in rural southern communities: practitioner perceptions of environmental opportunities and barriers. *The Journal of Rural Health*.

[34] Swaminathan S., Thomas T., Yusuf S., Vaz M. (2013). Clustering of diet, physical activity and overweight in parents and offspring in South India. *European Journal of Clinical Nutrition*.

[35] Martín-Matillas M., Ortega F. B., Chillon P. (2011). Physical activity among Spanish adolescents: relationship with their relatives’ physical activity - the AVENA study. *Journal of Sports Sciences*.

[36] Cheng L. A., Mendonça G., Farias Júnior J. C. d. (2014). Physical activity in adolescents: analysis of the social influence of parents and friends. *Jornal de Pediatria*.

[37] McGrath L. J., Hopkins W. G., Hinckson E. A. (2015). Associations of objectively measured built-environment attributes with youth moderate-vigorous physical activity: a systematic review and meta-analysis. *Sports Medicine*.

[38] Jones A. P., Coombes E. G., Griffin S. J., van Sluijs E. M. (2009). Environmental supportiveness for physical activity in English schoolchildren: a study using global positioning systems. *International Journal of Behavioral Nutrition and Physical Activity*.

[39] Coombes E., van Sluijs E., Jones A. (2013). Is environmental setting associated with the intensity and duration of children’s physical activity? findings from the SPEEDY GPS study. *Health & Place*.

[40] Wright M. S., Wilson D. K., Griffin S., Evans A. (2010). A qualitative study of parental modeling and social support for physical activity in underserved adolescents. *Health Education Research*.

[41] Ahola R., Pyky R., Jamsa T. (2013). Gamified physical activation of young men--a multidisciplinary population-based randomized controlled trial (MOPO study). *BMC Public Health*.

[42] Wennman H., Kronholm E., Partonen T. (2014). Physical activity and sleep profiles in Finnish men and women. *BMC Public Health*.

[43] Nigg C. R., Rossi J. S., Norman G. J., Benisovich S. V. (1998). Structure of decisional balance for exercise adoption. *Annals of Behavioral Medicine*.

[44] Raitasalo R. (2007). *Mood Questionnaire. Finnish Modification of the Short Form of the Beck Depression Inventory Measuring Depression Symptoms and Self-Esteem*.

[45] Dumith S. C., Gigante D. P., Domingues M. R., Hallal P. C., Menezes A. M., Kohl H. W. (2012). Predictors of physical activity change during adolescence: a 3·5-year follow-up. *Public Health Nutrition*.

[46] Schoeppe S., Röbl M., Liersch S., Krauth C., Walter U. (2016). Mothers and fathers both matter: the positive influence of parental physical activity modeling on children’s leisure-time physical activity. *Pediatric Exercise Science*.

[47] Official Statistics of Finland (OSF) (2021). http://www.stat.fi/til/perh/meta_en.html.

[48] Pietilä M., Neuvonen M., Borodulin K., Korpela K., Sievänen T., Tyrväinen L. (2015). Relationships between exposure to urban green spaces, physical activity and self-rated health. *Journal of Outdoor Recreation and Tourism*.

[49] Moore J. B., Brinkley J., Crawford T. W., Evenson K. R., Brownson R. C. (2013). Association of the built environment with physical activity and adiposity in rural and urban youth. *Preventive Medicine*.

[50] Moore J. B., Beets M. W., Morris S. F., Kolbe M. B. (2014). Comparison of objectively measured physical activity levels of rural, suburban, and urban youth. *American Journal of Preventive Medicine*.

[51] Euler R., Jimenez E. Y., Sanders S. (2019). Rural-urban differences in baseline dietary intake and physical activity levels of adolescents. *Preventing Chronic Disease*.

[52] Tyrväinen L., Mäkinen K., Schipperijn J. (2007). Tools for mapping social values of urban woodlands and other green areas. *Landscape and Urban Planning*.

[53] Flowers E. P., Freeman P., Gladwell V. F. (2016). A cross-sectional study examining predictors of visit frequency to local green space and the impact this has on physical activity levels. *BMC Public Health*.

[54] Ainsworth B. E., Levy S. S., Oja P., Borms J. (2004). Assessment of health-enhancing physical activity. Methodological issues. *Health Enhancing Physical Activity*.

[55] Tucker J. M., Welk G. J., Beyler N. K. (2011). Physical activity in U.S. adults. *American Journal of Preventive Medicine*.

